# Whole-exome sequencing of individuals from an isolated population implicates rare risk variants in bipolar disorder

**DOI:** 10.1038/tp.2017.3

**Published:** 2017-02-14

**Authors:** F Lescai, T D Als, Q Li, M Nyegaard, G Andorsdottir, M Biskopstø, A Hedemand, A Fiorentino, N O'Brien, A Jarram, J Liang, J Grove, J Pallesen, E Eickhardt, M Mattheisen, L Bolund, D Demontis, A G Wang, A McQuillin, O Mors, J Wang, A D Børglum

**Affiliations:** 1 Department of Biomedicine, Aarhus University, Aarhus, Denmark; 2 iPSYCH—The Lundbeck Foundation Initiative for Integrative Psychiatric Research, Aarhus, Denmark; 3 iSEQ—Centre for Integrative Sequencing, Aarhus University, Aarhus, Denmark; 4 BGI-Shenzhen, Shenzhen, China; 5 Genetic Biobank of the Faroe Islands, Tórshavn, Faroe Islands; 6 Division of Psychiatry, University College London, London, UK; 7 BiRC—Bioinformatics Research Centre, Aarhus University, Aarhus, Denmark; 8 Aarhus University Hospital, Risskov, Aarhus, Denmark; 9 Mental Health Centre Amager, Copenhagen, Denmark

## Abstract

Bipolar disorder affects about 1% of the world's population, and its estimated heritability is about 75%. Only few whole genome or whole-exome sequencing studies in bipolar disorder have been reported, and no rare coding variants have yet been robustly identified. The use of isolated populations might help finding variants with a recent origin, more likely to have drifted to higher frequency by chance. Following this approach, we investigated 28 bipolar cases and 214 controls from the Faroe Islands by whole exome sequencing, and the results were followed-up in a British sample of 2025 cases and 1358 controls. Seventeen variants in 16 genes in the single-variant analysis, and 3 genes in the gene-based statistics surpassed exome-wide significance in the discovery phase. The discovery findings were supported by enrichment analysis of common variants from genome-wide association studies (GWAS) data and interrogation of protein–protein interaction networks. The replication in the British sample confirmed the association with *NOS1* (missense variant rs79487279) and *NCL* (gene-based test). A number of variants from the discovery set were not present in the replication sample, including a novel *PITPNM2* missense variant, which is located in a highly significant schizophrenia GWAS locus. Likewise, *PIK3C2A* identified in the gene-based analysis is located in a combined bipolar and schizophrenia GWAS locus. Our results show support both for existing findings in the literature, as well as for new risk genes, and identify rare variants that might provide additional information on the underlying biology of bipolar disorder.

## Introduction

Bipolar disorder is a disturbance of mood in which patients display episodes of depression, often characterised by low mood, loss of pleasure and energy, and episodes of hypomania or mania, with irritable mood, increased energy and reduced sleep: this condition affects about 1% of the world's population.^1^ The estimated heritability of bipolar disorder is about 75%.^2^ There is a substantial overlap in genetic aetiology with schizophrenia with several loci influencing susceptibility to both disorders, for example, at *CACNA1C* and *PIK3C2A*.^3, 4, 5^ Analysis of data from common variants in genome-wide association studies (GWAS) has shown the genetic correlation between the two disorders to be as high as 0.68.^6^ Although common variants may explain a large proportion of the variance in liability to bipolar disorder (and other psychiatric disorders), a substantial part of the estimated heritability is still unaccounted for.^6, 7^ Rare risk variants not effectively assessed by GWAS may explain part of this hidden heritability.

Whole genome or whole-exome sequencing studies have proved successful in identifying rare variants for Mendelian disorders and more recently also for complex disorders.^8^ Among psychiatric disorders, in particular exome sequencing studies in autism have successfully identified rare transmitted variants and *de novo* mutations conveying large effect on disease risk.^9, 10^ In bipolar disorder, only few whole genome or whole-exome sequencing studies have been reported, most of which investigated large pedigrees, and no rare coding variants have yet been robustly identified.^11^

Owing to the increased genetic drift during founding, followed by population expansion, isolated populations may be particularly useful in identifying rare disease variants that may appear at higher frequencies compared with outbred populations,^12, 13, 14^ as has been shown previously for several monogenic^15, 16^ as well as some complex disorders.^17^ Variants with a recent origin are thus more likely to have drifted to higher frequency by chance in a smaller isolated population compared with a larger outbred population.^18^ Furthermore, isolated populations are relatively homogeneous in genetic background and environmental exposure, and control cohorts might reflect better the composition of the population they are drawn from, as they often represent a larger proportion of that population compared with controls from outbred populations.

The population of the Faroe Islands is an isolated population. It was founded by a small number of individuals in the nineth century and has experienced limited immigration for several centuries.^19^ Owing to the extensive founder and drift effects with rare variants drifting to increased frequencies, certain monogenic disorders appear at highly increased frequencies in the population.^20^ For instance, in the Faroese population glycogen storage disease III (GSD3, OMIM #232400) is caused by a single nonsense mutation that is >250 times more frequent than in outbred populations.^21^

We aimed to identify rare risk variants in bipolar disorder, by investigating whole-exome sequences of 28 individuals with bipolar disorder and 214 controls from the isolated population of the Faroe Islands. The results were followed-up in a British sample of 2025 bipolar disorder cases and 1358 controls, as well as by enrichment analysis of large GWAS data sets and by the analysis of significant physical connectivity among proteins encoded for by genes nominally significant in this study.

## Materials and Methods

### Faroese subjects

Patients were included from the Department of Psychiatry, National Hospital in Torshavn, the capital of the Faroe Islands. The patients included were interviewed by trained interviewers using a brief version of Present State Examination. Based on the hospital records and the interview, a clinical description was made of each patient by an experienced psychiatrist. The final diagnosis was made by best-estimate by an experienced psychiatrist (AGW) on the basis of all the material and records, according to ICD-10, Diagnostic Criteria for Research. All patients included had bipolar disorder according to ICD-10 and bipolar disorder type 1 according to DSM-IV. Controls were included by public advertising and assessed as having no psychiatric record and confirming this in a short interview.

Genomic DNA was purified from whole blood samples at the Faroese Genetic Biobank according to standard procedures used in the laboratories.

The study has been approved by the local scientific ethical committee of the Faroe Islands.

### British subjects

The UCL bipolar disorder subjects comprised 2025 subjects suffering from bipolar disorder type I (83%) or bipolar disorder type II. All subjects had been given a National Health Service (NHS) clinical diagnosis of ICD-10 bipolar disorder and then needed to fulfill the criteria for the lifetime version of the Schizophrenia and Affective Disorder Schedule (SADS-L)^22^ which provides a research diagnostic criteria (RDC) diagnosis.^23^ The UCL control sample comprised in total 1358 subjects. This included 878 subjects with no first-degree family or personal history of psychiatric illness, supplemented with an additional 480 unscreened normal British subjects obtained from the European Collection of Animal Cell Cultures (ECACC). The bipolar subjects and the screened controls underwent ancestral screening to be included only if at least three out of four grandparents were English, Scottish, Welsh or Irish and if the fourth grandparent was non-Jewish European. National Health Service multicenter research ethics approval was obtained. All participants provided signed consent. DNA samples were collected from blood or saliva samples and genomic DNA was purified using standard techniques.

### Sequence processing

#### Library and sequencing

The library preparation was performed according to the manufacturer's instructions, and the exome was captured using Agilent SureSelect version 3 (Agilent Technologies, Santa Clara, CA, USA). The libraries were sequenced on an Illumina HiSeq2500 (Illumina, San Diego, CA, USA).

#### Mapping

The sample reads were aligned to the genome (reference GRCh37) using BWA version 0.7.4 (http://bio-bwa.sourceforge.net), converted to BAM format and indexed using SAMtools (version 0.1.18, https://samtools.github.io). The samples were re-aligned, marked for duplicates and recalibrated using GATK^24^ and Queue (version 2.7-2, https://software.broadinstitute.org/gatk/) as pipeline manager.

#### Variant calling

The variants were called using HaplotypeCaller and UnifiedGenotyper, processed with VQSR following the best practices for the version in use, and pre-filtered by ‘PASS' at the output of each caller. The calls were merged by including, in order of priority, all ‘PASS' HaplotypeCaller variants and all ‘PASS' calls unique to UnifiedGenotyper. This implies that (i) in case of overlapping ‘PASS' variants, the calls from HaplotypeCaller were included, (ii) in case of overlapping variants filtered by VQSR in HaplotypeCaller and ‘PASS' in UnifiedGenotyper, the UnifiedGenotyper calls were included and (iii) all ‘PASS' non-overlapping variants unique to each Caller were included.

#### Annotation

The variants were annotated using the snpEFF (version 3.3 h, http://snpeff.sourceforge.net), EPACTS (version 3.3, http://genome.sph.umich.edu/wiki/EPACTS) and Variant Effect Predictor (version 75, http://www.ensembl.org/info/docs/tools/vep/index.html) tools from ENSEMBL, and the variant type using the GATK VariantAnnotator. On the basis of this, the variant calls were grouped into single-nucleotide polymorphisms (SNPs), insertions, deletions and multiallelelic calls. SIFT, Polyphen and Loftee have been used to annotate missense mutation with additional predictions about potentially damaging consequences. The group of multiallelic calls comprise the variant types identified by GATK as ‘MULTIALLELIC_COMPLEX.Other' and ‘MULTIALLELIC_MIXED': the first includes the variants represented by multiple alleles containing insertions or deletions (or a combination hereof) of different sizes, whereas the second includes the variants in which multiple alternative alleles can be a combination of SNPs, insertions and/or deletions.

### Genotyping and validation

Genotyping of the UCL sample and genotype validation on Faroese samples was performed using the Sequenom MassARRAY iPLEX technology^25^ or Fluidigm technology.^26^ An in-house Perl script was used to process the VCF file with the significant variants and format the polymorphisms according to Sequenom and Fluidigm requirements. The output for Sequenom was used to design the amplicon and extension primers using the Sequenom Assay Design Suite version 1.0 (Sequenom, San Diego, CA, USA) with high multiplexing iPLEX presets. The genotyping was performed according to manufacturer's standard protocols for iPLEX. The variants that failed Sequenom design or genotyping were typed with Fluidigm: the output of our scripts was processed with D3 Assay Design, and the samples were processed according to Fluidigm. In both cases all genotyping results were manually checked to verify the cluster plots.

### Statistical analyses

Pairwise coefficients of Identity By Descent and kinship coefficients (*k*_ij_) was estimated using the method of moments approach^27^ as implemented in the R-package SNPRelate,^28^ to identify unknown relationships and confirming known first-degree relationships. These analyses were conducted on a filtered set of single-nucleotide variants (SNVs), filtered by LD-pruning (*r*^2^<0.002), a missing rate <0.005 and a minor allele frequency >0.01. The cryptic relatedness evident from the results of these analyses (results not shown), was corrected for in the association analysis, by adopting the EMMAX^29^ approach as implemented in EPACTS and suggested previously in the literature.^12^

The primary analyses of the Faroese variants data were performed using EPACTS v.2.6. 4 samples have been excluded by EPACTS QC filters (1 case, 3 controls), resulting in a final dataset of 27 cases and 211 controls ready for the analysis. To analyse the single variants according to our hypothesis, we filtered for all called variants either novel or by frequency lower than 0.05 in 1000 Genomes (CEU+GBR) and present in at least 3 individuals in our dataset. Subsequently, the single-variant analysis on the selected subset was performed using the ‘q.emmax' statistics,^29^ to account for any hidden relatedness of the sample. This method was chosen because of its ability to handle related individuals. The method has been originally developed for quantitative traits, but can be applied to binary traits in the spirit of Armitage trend test giving reliable *P*-values but potentially inaccurate effect measures, which we therefore ignore. This is not an exact method but based on asymptotic approximations that may not be accurate when there are small cell counts.

For the gene-burden statistics, the ‘Emmax CMC-like' method was employed.^29, 30^ To collapse the rare variants into gene-loci, the variants were selected by allowing a maximum minor allele frequency (calculated on the entire sample) of 5%, and having the following consequences, as annotated by VEP: transcript_ablation, splice_donor_variant, splice_acceptor_variant, stop_gained, frameshift_variant, stop_lost, initiator_codon_variant, inframe_insertion, inframe_deletion, missense_variant, transcript_amplification, splice_region_variant, incomplete_terminal_codon_variant.

The statistical analyses of the genotypes from the replication phase for the single-marker analysis were conducted using Fisher's exact test, as implemented in PLINK 1.9.^18^

The replication of the gene-based tests from the British population was performed using the AssotesteR package in R (http://cran.r-project.org/package=AssotesteR), and choosing the classic CMC test, as the approach implemented on EPACTS can only be used with a larger genome/exome-wide dataset. To account for multiple testing, empirical *P*-values have been calculated with 1000 permutations, as implemented in the R-package.

For the risk gene enrichment analysis we used MAGMA^31^ and default settings, as well as INRICH^32^ with default settings, using top-1% GWAS results. INRICH uses a permutation procedure on genomic intervals, whereas MAGMA is based on a multiple regression model. We used publicly available summary statistics from single-marker GWASs^33^ considering only variants outside the broad MHC-region (chr6:25M-35M) and filtered for info score ⩾0.8. Genes were annotated using Ensemble (GRCh37.p13). Information about the genetic correlation pattern in the data (linkage disequilibrium) was obtained using the 1000 Genomes European panel.^34^

For the protein–protein interaction analysis DAPPLE was used, which looks for significant physical connectivity among proteins encoded by the genes associated in the study. DAPPLE builds interaction networks from proteins encoded by the genes reported in the association study and connected either by direct interactions (that is, when both were significantly associated) or indirect interactions (that is, through proteins not resulting from the association analysis). To identify candidate loci, a score is calculated for each gene by enumerating the number of its connections and comparing this number to the values obtained in permuted networks (50 000 permutations).^35^ The *P*-value of this test is the one we refer to in the description of this study.

## Results

Whole exomes from 28 bipolar cases and 214 controls were sequenced at an average depth of 35 × . After mapping the sequences to the GRCh37 version reference of the human genome, the bam files were processed using GATK (see ‘Materials and Methods' section), and a total of 259 904 variants were called. These included 230 797 SNVs, 10 029 insertions and 13 198 deletions: among those we annotated 2575 loss-of-function (LoF) variants and 54 967 missense mutations. Overall, 47 800 of the called variants were novel, and were not present in dbSNP.

### Single variant analysis

We designed the study to target rare risk variants (that might have increased in frequency in the isolated Faroese population) and, consequently, we included only variants at frequencies lower than 0.05 (or not present) in the 1000 Genomes data (CEU and GBR samples, release 20110521) (Supplementary Figure 1). In addition, given the small discovery sample size, we limited the analysis to those variants appearing in more than 2 individuals (allele count >2). This filtering strategy produced an analysis-ready dataset of 86 563 variants, corresponding to an experiment/exome-wide significance threshold of 5.78 × 10^−7^ for single-variant association after Bonferroni correction.

**Figure 1:**
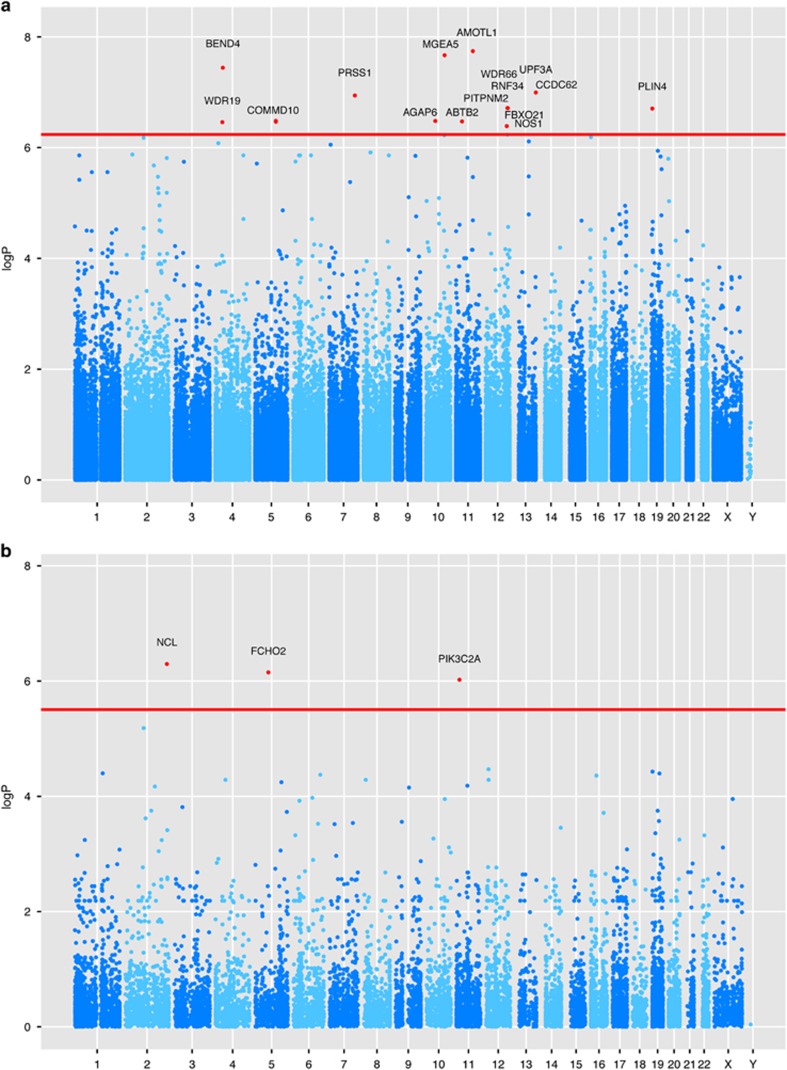
The figures report the Manhattan plots of the single-variant analysis with q.emmax (**a**) and gene-based CMC-like emmax (**b**) as implemented in EPACTS. The horizontal red line indicates the significant thresholds (*P*-value threshold of 5.78 × 10^−7^ for single-marker tests, and 3.12 × 10^−6^ for the gene-based tests). Significant variants are annotated with their corresponding gene from ENSEMBL data.

For association analysis we used the mixed model method q.emmax,^29^ implemented in the software package EPACTS, to adjust for relatedness and population structure within the sample. This analysis resulted in 17 variants in 16 genes surpassing exome-wide significance (Figure 1a). We decided to follow-up all variants with a nominal *P*-value <10^−6^ adding up to a total of 24 variants from 18 loci (Supplementary Table 1 and Supplementary Table 2).

As both common and rare risk variants often occur in the same genes/loci,^36, 37^ we investigated whether the identified top-18 loci showed risk enrichment for common variants in large bipolar and schizophrenia GWAS data sets,^33, 38^ using the two methods INRICH and MAGMA.^31, 32^ Interestingly, we found evidence of enrichment for schizophrenia risk by INRICH (*P*-value=0.031) assessing the top-1% associated SNPs, and by MAGMA (*P*=0.052), supporting the validity of the identified rare variants as a group. This enrichment reflects that several of the identified rare variants are located in loci showing *P*-values in the order of 10^−4^ to 10^−6^ in the GWAS (Table 1). Noteworthy, the *PITPNM2* missense variant is located in a genome-wide significant locus, and it is only described in the ExAC database (http://exac.broadinstitute.org/) with a frequency of 0.0006793. Furthermore, the observed enrichment indicates that some of the rare variants identified may influence susceptibility to both bipolar disorder and schizophrenia.

We did not observe any enrichment for bipolar common variant risk, which may be due to a substantially smaller sample size than is the case for the schizophrenia GWAS, providing relatively low power for the enrichment analysis in bipolar disorder.

We next followed-up the top-24 rare variants by genotyping a British sample of 2025 bipolar cases and 1358 controls. We chose a British sample as the Faroese population was founded partly by individuals from the British Isles and partly by Scandinavian Vikings,^40^ suggesting that risk variants may be shared among the British and Faroese populations.^41^ Fifteen variants from 13 loci were successfully genotyped; 9 were either not present in the British cohort or failed genotyping (Supplementary Table 1). The *NOS1* missense variant was the only variant showing significant association withstanding Bonferroni correction (*P*=0.002, *P*_corrected_=0.032; Table 1).

### Gene-wise analysis

To perform collapsing statistics on the rare variants, we collapsed into gene-loci those variants with predicted significant biological effects on the coding regions (see ‘Materials and Methods' section) and a maximum minor allele frequency of 5% in the whole dataset. The CMC-like burden test^30^ resulted in 419 nominally significant genes at *P*<0.01, and three genes significant exome-wide after Bonferroni correction (Figure 1b) and including more than one rare variant.

In an attempt to replicate the results, we genotyped all variants that contributed to the three significant gene-based tests in the British sample (Supplementary Table 3). However, only in the case of *NCL* were all variants present in the British sample. Thus, a regular replication test could be performed solely for *NCL*, which showed significant association (*P*=0.029, Table 1).

**Figure 2:**
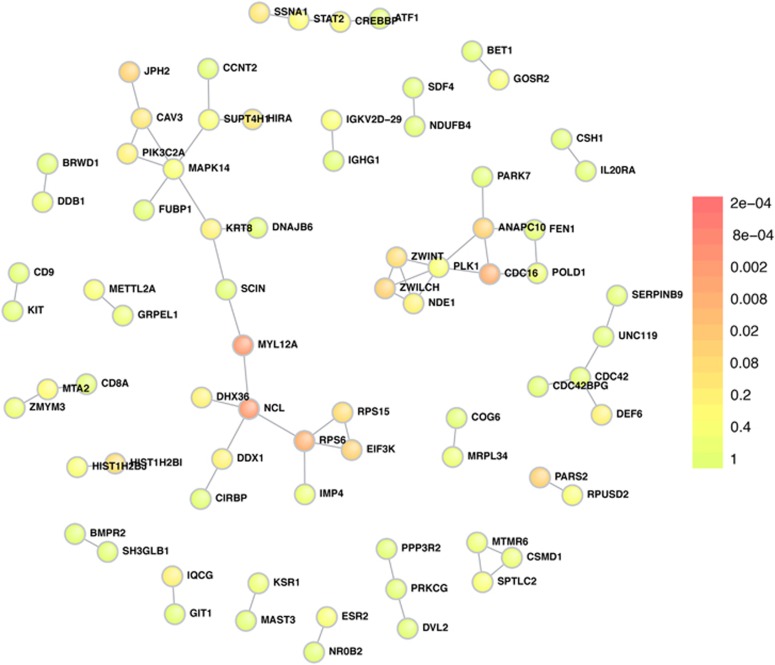
The plot shows the most significant connections resulting from a DAPPLE analysis of the 419 nominally significant genes (*P*-value <0.01) in the CMC-like emmax burden test in EPACTS of the Faroese sample. The colour code indicates the corrected *P*-value range of the DAPPLE analysis (seed scores).

On the nominally significant burden associations (419 genes at *P*-value <0.01), we performed a DAPPLE analysis,^35^ investigating whether the genes implicate a limited set of underlying mechanisms detectable by protein–protein interactions. The analysis supported 16 of the genes from the gene-burden analysis, in terms of protein–protein connectivity (Supplementary Table 4). Remarkably, the most significant gene in this analysis was *NCL*, with a corrected *P*-value of 0.002 (Figure 2), which supports the observed association of *NCL* with bipolar disorder, and suggests that more members of its interaction network might be implicated in disease susceptibility.

## Discussion

Targeting rare risk variants we sought to take advantage of using an isolated population, in which some of the variants that are very rare in outbred populations have been found highly increased in frequency, including mutations for rare Mendelian disorders. Risk alleles identified in isolated populations may, however, either be extremely rare in other populations or appear private and not observed elsewhere.^17, 42^ Findings using isolated populations may therefore not necessarily generalise to other populations, thus making replication difficult. To reduce this limitation, we selected a related British population for replication analysis, and also sought more indirect support of the findings via enrichment analysis of common variants from GWAS data and interrogation of protein–protein interaction networks within the discovery data set. Although the discovery sample was very limited in size, and thus prone to yield spurious findings, the study identified significant associations with single variants and genes that could be replicated in the follow-up sample and/or were supported by other lines of evidence.

Among the most interesting findings, the *NOS1* missense variant is intriguing. The variant is classified by SIFT as ‘deleterious' on transcript ENST00000338101, and while predicted as ‘benign' by Polyphen it is classified as ‘probably damaging' by LoFtool (Supplementary Table 2, for more details). This polymorphism shows exome-wide significance in the Faroese population and replicates in the British sample. *NOS1* encodes the neuronal nitric oxide (NO) synthase. NO is a gaseous neurotransmitter thought to have important roles in several behavioural domains. It acts as the second messenger of the *N*-methyl-d-aspartate receptor and interacts with both the dopaminergic as well as the serotonergic system.^43, 44^ Investigations of animal models and human genetic studies have implicated *NOS1* with both mood disorders and schizophrenia but with partially conflicting results.^44, 45^ Notably, the *NOS1* locus yields *P*-values of 10^−6^ in the most recent schizophrenia GWAS.^33^ Our findings support a role of this enzyme in bipolar disorder susceptibility and suggest the identified rare missense variant as a causal variant, providing a good basis for functional studies which relevance is further highlighted by the existence of numerous pharmaceutical targets for the mechanisms of action of NO.^44, 46^

A synonymous variant in *FBXO21*, a gene that neighbours *NOS1*, showed similar associations. No apparent evidence from the literature seems to implicate *FBXO21* with mental disorders, and the observed association is probably due to the strong linkage disequilibrium with the *NOS1* variant (*r*^2^=0.897).

The identified *PITPNM2* missense variant was only seen in the Faroese population. *PITPNM2* encodes a phosphatidylinositol transfer protein with limited functional information. It is located in a highly significant schizophrenia GWAS locus that harbours multiple genes.^33^ Thus, the present results not only suggest *PITPNM2* to be involved in bipolar disorder but also point to the gene as the causal culprit in this multi-gene schizophrenia locus.

Although the *P2RX7* variants fell just below the significance threshold and did not replicate in the British sample, it is worth noting that the gene has previously been associated with bipolar disorder,^39, 47^ including a study of a British sample overlapping with the present sample, showing association with another *P2RX7* variant.^8^ The gene belongs to a family of purinoreceptors for ATP, which function as ligand-gated ion-channels, and seem to have a role in the ATP-induced glutamate transmission in the hippocampus.^48^

The gene-based analysis highlighted the *NCL* gene, showing exome-wide significance and replication in the British sample. NCL and part of its interaction network is involved in the synthesis and maturation of ribosomes.^49^ Ribosomal DNA transcription appears to be decreased in specific cortical layers of post mortem brains in unipolar depression but not bipolar disorder.^50^ Interestingly, this gene was also significant in our DAPPLE analysis. Furthermore, the NCL interaction network emphasized by the DAPPLE analysis included PIK3C2A, which too surpassed exome-wide significance in the Faroese population and is supported by genome-wide significance in GWAS combining bipolar disorder and schizophrenia.^4^ Both *PIK3C2A* and *PITPNM2* are part of the phosphatidylinositol pathway, which have been widely implicated in mental disorders such as bipolar, depression and schizophrenia^4, 51^

Finally, the String Database^52^ identifies an indirect interaction of NCL, through MDM2 with CREBBP. This transcription factor is also in DAPPLE output, although not significant in terms of connectivity, and its pathway has been connected to mental illnesses in a large body of literature.^53^ This might open opportunities to further investigate the regulation of transcription by these proteins in brain cells.

Summarizing, our results show support both for existing findings in the literature of bipolar disorder as well as for new risk genes in the disease aetiology. In particular, we identify rare variants that may provide direct leads informing on the underlying biology of bipolar disorder and schizophrenia.

## Figures and Tables

**Table 1:** Selected single variant and gene-burden results.

*Gene*	*Marker*	*Consequence*	*P-value Faroese*	*P-value UK (corrected)*	*Support in GWAS*
*NOS1*	rs79487279	missense_variant	4.09E−07	0.002 (0.032)	The *NOS1* locus has a lead signal in schizophrenia with *P*-value of 1.24 × 10^−6^ (rs2293052)^33^
*PITPNM2*	12:123489064_C/A	missense_variant	1.93E−07	NA	In highly significant schizophrenia locus with a lead *P*-value of 2.19 × 10^−14^ (rs2851447)^33^
*NCL*	Gene test	—	5.04E−07	0.016 (0.029)	—
*PIK3C2A*	Gene test	—	9.46E−07	NA	*PIK3C2A* has a significant lead signal with *P*-value of 6.46 × 10^−9^ in a combined bipolar and schizophrenia GWAS (rs4356203)^39^

The table summarises the most significant results of the study, both from single-variant analysis and gene-based tests. We report only results exome-wide significant in the Faroese population after Bonferroni correction (nominal *P*-values thresholds of 5.78 × 10^−7^ for the single variants and 3.12 × 10^−6^ for the gene tests). The *P*-value for the UK sample was calculated with a Fisher exact test on the genotype model with PLINK 1.9, and we report in parenthesis the *P*-value after Bonferroni correction. In the last column we indicate whether our finding is supported by genome-wide association studies (GWAS).
