# Supplementary material for: Whole-exome sequencing of individuals from an isolated population implicates rare risk variants in bipolar disorder
Source: Transl Psychiatry. 2017 Feb 14;7(2):e1034–. doi: 10.1038/tp.2017.3 (PMC5438033; doi:10.1038/tp.2017.3)
Supplement: Supplementary Table [file tp20173x1.docx]

**Supplementary Table 1 – Single variant analysis**

| **Marker** | **Chr** | **Position^+^** | **Gene** | **Consequence** | **MAF Faroese** | **AC Faroese** | **CTRL Faroese** | **CASE Faroese** | **P-value Faroese** | **MAF UK** | **AC UK** | **CTRL UK** | **CASE UK** | **P-value UK (bonferroni)** | **Validation^#^** |
| --- | --- | --- | --- | --- | --- | --- | --- | --- | --- | --- | --- | --- | --- | --- | --- |
| *rs2228139** | *2* | *102781649* | *IL1R1* | *missense_variant* | *0.02731* | *13* | 206/5/0 | 21/4/2 | *6.65E-07* | *0.06924* | *389* | 1425/199/12 | 927/158/4 | *0.104 (1)* | *validated* |
| *rs7690911** | *4* | *16899946* | *LDB2* | *nc_transcript_variant* | *0.02101* | *10* | 207/4/0 | 21/6/0 | *8.31E-07* | *0.07137* | *475* | 1731/253/13 | 1120/184/6 | *0.4074 (1)* | *validated* |
| rs187546086 | 4 | 39254828 | *WDR19* | missense_variant | 0.0105 | 5 | 210/1/0 | 23/4/0 | 3.47E-07 | 0.009874 | 54 | 1744/36/0 | 1106/18/0 | 0.4816 (1) | validated |
| rs144453545 | 4 | 42122289 | *BEND4* | missense_variant | 0.01891 | 9 | 208/3/0 | 21/6/0 | 3.61E-08 | 0.02352 | 151 | 1914/93/1 | 1272/56/0 | 0.764 (1) | validated |
| rs41296577 | 5 | 115426932 | *COMMD10* | intron_variant | 0.01059 | 5 | 208/1/0 | 23/4/0 | 3.40E-07 | 0.03645 | 198 | 1510/110/2 | 992/82/1 | 0.741 (1) | validated |
| rs17138918 | 5 | 115469668 | *COMMD10* | intron_variant | 0.0105 | 5 | 210/1/0 | 23/4/0 | 3.29E-07 | 0.03271 | 218 | 1879/119/1 | 1237/95/1 | 0.3426 (1) | validated |
| *7:7421241_C/T** | *7* | *7421241* | *COL28A1* | *splice_region_variant* | *0.0063* | *3* | 211/0/0 | 24/3/0 | *8.85E-07* | *0.0004347* | *1* | 2003/0/0 | 1338/1/0 | *0.4007 (1)* | *validated* |
| 7:142460482_G/A,T | 7 | 142460482 | *PRSS1* | intron_variant | 0.02941 | 462 | 0/5/206 | 4/1/22 | 1.14E-07 |  |  |  |  | NA | assay failed |
| 10:51754236_A/T | 10 | 51754236 | *AGAP6* | intron_variant | 0.01471 | 7 | 209/2/0 | 22/5/0 | 3.30E-07 |  |  |  |  | NA | assay failed |
| *10:101802338_A/G** | *10* | *101802338* | *CPN1* | *splice_region_variant* | *0.0063* | *3* | 211/0/0 | 24/3/0 | *5.98E-07* |  |  |  |  | *NA* | *monomorphic* |
| rs200472428 | 10 | 103569924 | *MGEA5* | intron_variant | 0.0084 | 4 | 211/0/0 | 23/4/0 | 2.14E-08 | 0.006137 | 40 | 1978/18/0 | 1306/22/0 | 0.0726 (1) | validated |
| rs147573517 | 11 | 34175792 | *ABTB2* | intron_variant | 0.0063 | 3 | 211/0/0 | 24/3/0 | 3.36E-07 | 0.0116 | 79 | 1951/50/0 | 1310/29/0 | 0.5633 (1) | validated |
| rs192694640 | 11 | 94501751 | *AMOTL1* | intron_variant | 0.0084 | 4 | 211/0/0 | 23/4/0 | 1.81E-08 | 0.004653 | 27 | 1992/12/0 | 1313/15/0 | 0.1142 (1) | validated |
| rs117905145 | 12 | 117615374 | *FBXO21* | synonymous_variant | 0.01681 | 8 | 209/2/0 | 22/4/1 | 4.09E-07 | 0.004054 | 25 | 1981/22/0 | 1338/3/0 | 0.003429 (0.051435) | validated |
| **rs79487279** | **12** | **117705934** | ***NOS1*** | **missense_variant** | **0.01681** | **8** | 209/2/0 | 22/4/1 | **4.09E-07** | **0.004342** | **26** | 1982/23/0 | 1339/3/0 | **0.002138 (0.03207)** | validated |
| *rs28360446** | *12* | *121600140* | *P2RX7* | *3_prime_UTR_variant* | *0.01681* | *8* | 209/2/0 | 22/4/1 | *5.86E-07* | *0.0252* | *172* | 1894/110/0 | 1279/60/1 | *0.156 (1)* | *validated* |
| *rs76481706** | *12* | *121603294* | *P2RX7* | *intron_variant* | *0.01681* | *8* | 209/2/0 | 22/4/1 | *5.86E-07* | *0.02655* | *180* | 1880/114/1 | 1261/64/0 | *0.3751 (1)* | *validated* |
| 12:121837914_G/T | 12 | 121837914 | *RNF34* | 5_prime_UTR_variant | 0.0105 | 5 | 211/0/0 | 23/3/1 | 1.93E-07 |  |  |  |  | NA | assay failed |
| 12:122400101_G/A | 12 | 122400101 | *WDR66* | nc_transcript_variant | 0.0105 | 5 | 211/0/0 | 23/3/1 | 1.93E-07 |  |  |  |  | NA | monomorphic |
| 12:123290890_C/T | 12 | 123290890 | *CCDC62* | intron_variant | 0.0105 | 5 | 211/0/0 | 23/3/1 | 1.93E-07 |  |  |  |  | NA | monomorphic |
| 12:123489064_C/A | 12 | 123489064 | *PITPNM2* | missense_variant | 0.0105 | 5 | 211/0/0 | 23/3/1 | 1.93E-07 |  |  |  |  | NA | monomorphic |
| *rs8192737** | *13* | *76123901* | *RP11-29G8.3* | *non_coding_exon_variant* | *0.15126* | *72* | 168/36/7 | 10/12/5 | *7.73E-07* |  |  |  |  | *NA* | *assay failed* |
| rs146464930 | 13 | 115067419 | *UPF3A* | synonymous_variant | 0.01471 | 7 | 209/2/0 | 22/5/0 | 1.01E-07 | 0.02671 | 152 | 1695/74/3 | 1047/60/6 | 0.07512 (1) | validated |
| rs202096657 | 19 | 4504701 | *PLIN4* | missense_variant | 0.00682 | 3 | 196/0/0 | 21/3/0 | 1.97E-07 |  |  |  |  | NA | assay failed |

Results of the single variant analysis, performed on the Faroese sample with the q.emmax method in EPACTS (to account for hidden relatedness and population structure) and on the British sample with Fisher’s exact test (in parenthesis the values Bonferroni corrected for 15 independent tests). The * represents variants not exome-wide significant in the Faroese after Bonferroni correction.

^+^ position according to GRCh37

^#^ experimental validation by Sequenom or Fluidigm technology.

**Supplementary Table 2 – Consequence analysis for missense variants**

| **Marker** | **Chromosome** | **Position** | **Gene** | **Consequence** | **SIFT** | **Polyphen** | **LoFtool** |
| --- | --- | --- | --- | --- | --- | --- | --- |
| rs202096657 | 19 | 4504701 | PLIN4 | missense_variant | 0.3 (benign) | 0.041 (benign) | 0.888 (benign) |
| 12:123489064_C/A | 12 | 123489064 | PITPNM2 | missense_variant | 0.9 (tolerated) | (unknown) | 0.163 (probably damaging) |
| rs144453545 | 4 | 42122289 | BEND4 | missense_variant | 0.06 (tolerated) | 0.084 (benign) | 0.146 (probably damaging) |
| rs187546086 | 4 | 39254828 | WDR19 | missense_variant | 0.37 (benign) | 0.014 (benign) | 0.548 (possibly damaging) |
| rs79487279 | 12 | 117705934 | NOS1 | missense_variant | 0.03 (deleterious) | 0.022 (benign) | 0.306 (probably damaging) |
| *rs2228139** | *2* | *102781649* | *IL1R1* | *missense_variant* | *0.41 (tolerated)* | *0.001 (benign)* | *0.685 (benign)* |

**Supplementary Table 2 legend:**

The table reports the potential impact of the missense variants on the protein, as calculated by three different tools: SIFT (based on the degree of conservation of residues in sequence alignments), Polyphen (based on a machine learning classifier of protein structure and function data), and LoFtool (predicting susceptibility to disease based on genic intolerance and ratio of loss-of-function to synonymous mutations). The * represents variants not exome-wide significant in the Faroese after Bonferroni correction.

**Supplementary Table 3 – Gene-based analysis**

| **Gene** | **Contributing variants in Faroese** | **P-value Faroese** | **Variants present in UK sample** | **P-value UK (empirical)** |
| --- | --- | --- | --- | --- |
| NCL | rs139777351, rs66781921, rs117088491 | 5.04E-07 | rs139777351, rs66781921, rs117088491 | 0.016 (0.029) |
| FCHO2 | rs143520046, 5:72347239_C/G, rs185435, 5:72350365_G/A , 5:72374117_A/T | 7.04E-07 | rs143520046, rs185435* | 0.295 (0.313)* |
| PIK3C2A | rs199935176 , rs11604561 , 11:17134132_C/T , rs61733866, 11:17169069_G/A , rs61755370 | 9.46E-07 | rs199935176 , rs11604561, rs61733866, rs61755370* | 0.770 (0.754)* |

**Supplementary Table 3 legend**

The table shows the results of the CMC-like burden test performed with EPACTS in the Faroese sample, and the corresponding follow-up in the British sample, performed with CMC-test in the R Package “AssotesteR”. In order to account for multiple testing, empirical p-values have been calculated with 1000 permutations, as implemented in the R package.

* denotes that only a subset of the variants contributing to the Faroese signal are present in the UK sample.

**Supplementary Table 4**

| **GENE** | **P_uncorrected** | **P_corrected** |
| --- | --- | --- |
| NCL | 0.002101944 | 0.002101944 |
| HERC3 | 0.002101944 | 0.002101944 |
| MYL12A | 0.002101944 | 0.002101944 |
| CDC16 | 0.006299197 | 0.006299197 |
| RPS6 | 0.006299197 | 0.006299197 |
| FBXL20 | 0.006299197 | 0.006299197 |
| ALDH4A1 | 0.01675363 | 0.01675363 |
| CSH2 | 0.022999753 | 0.022999753 |
| JPH2 | 0.025077372 | 0.025077372 |
| PARS2 | 0.035432292 | 0.035432292 |
| ANAPC10 | 0.035432292 | 0.035432292 |
| EIF3K | 0.035432292 | 0.035432292 |
| LGALS13 | 0.037496641 | 0.037496641 |
| ZWILCH | 0.041618707 | 0.041618707 |
| CDKN1B | 0.047785219 | 0.047785219 |
| RPS15 | 0.05188517 | 0.05188517 |
| PGAM2 | 0.053931829 | 0.053931829 |
| CMIP | 0.055976276 | 0.055976276 |
| ZWINT | 0.055976276 | 0.055976276 |
| GLRX5 | 0.062096349 | 0.062096349 |
| HIST1H2BI | 0.06616534 | 0.06616534 |
| ZNF324B | 0.074276787 | 0.074276787 |
| ZNF74 | 0.07629912 | 0.07629912 |
| HIRA | 0.084366337 | 0.084366337 |
| SSNA1 | 0.086377613 | 0.086377613 |
| CLIC4 | 0.088386678 | 0.088386678 |
| CAV3 | 0.092398173 | 0.092398173 |
| FIG4 | 0.094400603 | 0.094400603 |
| ZNF329 | 0.094400603 | 0.094400603 |
| PIK3C2A | 0.137894584 | 0.137894584 |
| DHX36 | 0.143742654 | 0.143742654 |
| NDE1 | 0.155379085 | 0.155379085 |
| TDRD1 | 0.159240204 | 0.159240204 |
| KRT8 | 0.165015297 | 0.165015297 |
| ALDH3B1 | 0.165015297 | 0.165015297 |
| ZEB2 | 0.180318244 | 0.180318244 |
| DEF6 | 0.182221161 | 0.182221161 |
| IQCG | 0.189810714 | 0.189810714 |
| DDX1 | 0.19547966 | 0.19547966 |
| NFKBIE | 0.221671582 | 0.221671582 |
| CREBBP | 0.24377682 | 0.24377682 |
| RANBP6 | 0.247430067 | 0.247430067 |
| BAIAP2L1 | 0.254710024 | 0.254710024 |
| TBCE | 0.254710024 | 0.254710024 |
| VDAC1 | 0.261954598 | 0.261954598 |
| MTA2 | 0.263760213 | 0.263760213 |
| AGL | 0.29764673 | 0.29764673 |
| STAT2 | 0.29764673 | 0.29764673 |
| ESR2 | 0.301167292 | 0.301167292 |
| MAPK14 | 0.308181879 | 0.308181879 |
| THRAP3 | 0.308181879 | 0.308181879 |
| MTMR11 | 0.309929998 | 0.309929998 |
| METTL2A | 0.318637419 | 0.318637419 |
| SPTLC2 | 0.327289554 | 0.327289554 |
| HIST1H2BJ | 0.342724079 | 0.342724079 |
| SUPT4H1 | 0.346129648 | 0.346129648 |
| CASQ2 | 0.346129648 | 0.346129648 |
| THRB | 0.347829116 | 0.347829116 |
| PLK1 | 0.371389461 | 0.371389461 |
| RPUSD2 | 0.374719842 | 0.374719842 |
| GOSR2 | 0.384657912 | 0.384657912 |
| GPS1 | 0.399415746 | 0.399415746 |
| IGKV2D-29 | 0.399415746 | 0.399415746 |
| DDB1 | 0.409154789 | 0.409154789 |
| TFAP2A | 0.412383445 | 0.412383445 |
| C10orf88 | 0.417209844 | 0.417209844 |
| PRDM2 | 0.439469881 | 0.439469881 |
| MRPL34 | 0.439469881 | 0.439469881 |
| PODXL2 | 0.441043298 | 0.441043298 |
| PDLIM2 | 0.445750281 | 0.445750281 |
| GRPEL1 | 0.447314853 | 0.447314853 |
| DUOX1 | 0.450437361 | 0.450437361 |
| ANKFN1 | 0.458204933 | 0.458204933 |
| Sep-01 | 0.464379186 | 0.464379186 |
| TFAP4 | 0.464379186 | 0.464379186 |
| CSMD1 | 0.46591722 | 0.46591722 |
| CAPN6 | 0.468986655 | 0.468986655 |
| KSR1 | 0.478141886 | 0.478141886 |
| IMP4 | 0.479660018 | 0.479660018 |
| ZMYM3 | 0.485710432 | 0.485710432 |
| MTMR6 | 0.487217506 | 0.487217506 |
| POLD1 | 0.49471971 | 0.49471971 |
| HOXC4 | 0.502166627 | 0.502166627 |
| MICAL1 | 0.511029952 | 0.511029952 |
| LANCL3 | 0.516894608 | 0.516894608 |
| PEX5L | 0.527072615 | 0.527072615 |
| C1R | 0.532839968 | 0.532839968 |
| OPN1MW2 | 0.548517748 | 0.548517748 |
| HTR6 | 0.548517748 | 0.548517748 |
| TP53RK | 0.549929732 | 0.549929732 |
| FEN1 | 0.561145996 | 0.561145996 |
| ATF1 | 0.563927948 | 0.563927948 |
| NR0B2 | 0.56808429 | 0.56808429 |
| SLC13A2 | 0.57222073 | 0.57222073 |
| SCIN | 0.576337266 | 0.576337266 |
| HCLS1 | 0.577705022 | 0.577705022 |
| ZDHHC23 | 0.583153933 | 0.583153933 |
| SERPINB9 | 0.59662141 | 0.59662141 |
| PPP3R2 | 0.599288369 | 0.599288369 |
| SH3GLB1 | 0.601946482 | 0.601946482 |
| ZNF609 | 0.604595749 | 0.604595749 |
| IP6K3 | 0.607236171 | 0.607236171 |
| NHP2 | 0.612490477 | 0.612490477 |
| CCNT2 | 0.613798525 | 0.613798525 |
| KIT | 0.621600374 | 0.621600374 |
| HTR1F | 0.621600374 | 0.621600374 |
| DHX30 | 0.643273283 | 0.643273283 |
| CD9 | 0.650769957 | 0.650769957 |
| NPAT | 0.658187021 | 0.658187021 |
| MRPL39 | 0.659415458 | 0.659415458 |
| BRWD1 | 0.661865699 | 0.661865699 |
| EMCN | 0.667952601 | 0.667952601 |
| BET1 | 0.672782317 | 0.672782317 |
| TSPAN6 | 0.681149181 | 0.681149181 |
| PSMD3 | 0.681149181 | 0.681149181 |
| SLC27A4 | 0.691747355 | 0.691747355 |
| GRB14 | 0.69407818 | 0.69407818 |
| ARG1 | 0.698713292 | 0.698713292 |
| PGM2 | 0.709012927 | 0.709012927 |
| AHDC1 | 0.709012927 | 0.709012927 |
| SMARCAD1 | 0.716899915 | 0.716899915 |
| SYNE2 | 0.720246882 | 0.720246882 |
| CIRBP | 0.730168366 | 0.730168366 |
| URB1 | 0.745245748 | 0.745245748 |
| TRPV6 | 0.747364278 | 0.747364278 |
| SPATA2 | 0.748420225 | 0.748420225 |
| SATB2 | 0.750525486 | 0.750525486 |
| WTAP | 0.768063061 | 0.768063061 |
| NIPBL | 0.779070346 | 0.779070346 |
| GRIK1 | 0.785935663 | 0.785935663 |
| TECPR1 | 0.786907578 | 0.786907578 |
| G6PD | 0.790773119 | 0.790773119 |
| COG6 | 0.792692622 | 0.792692622 |
| DNAJB6 | 0.794603279 | 0.794603279 |
| SDF4 | 0.801220919 | 0.801220919 |
| IL1R1 | 0.804953776 | 0.804953776 |
| SRR | 0.805881462 | 0.805881462 |
| ZNF691 | 0.812313343 | 0.812313343 |
| KCNH2 | 0.81774014 | 0.81774014 |
| SEC61A1 | 0.81774014 | 0.81774014 |
| CDC42 | 0.821313776 | 0.821313776 |
| PARK7 | 0.823087325 | 0.823087325 |
| CD7 | 0.824852029 | 0.824852029 |
| WDR12 | 0.826607887 | 0.826607887 |
| DTX3L | 0.846164478 | 0.846164478 |
| GALK2 | 0.851884286 | 0.851884286 |
| RPRD2 | 0.856700733 | 0.856700733 |
| SOX6 | 0.856700733 | 0.856700733 |
| WNK1 | 0.857495735 | 0.857495735 |
| ELF4 | 0.859079103 | 0.859079103 |
| SLCO1A2 | 0.860653626 | 0.860653626 |
| ZMIZ1 | 0.862998825 | 0.862998825 |
| CSH1 | 0.868393556 | 0.868393556 |
| ELMO2 | 0.870672412 | 0.870672412 |
| IL20RA | 0.870672412 | 0.870672412 |
| MRPS7 | 0.872180592 | 0.872180592 |
| UNC119 | 0.872931366 | 0.872931366 |
| MRPL9 | 0.877389565 | 0.877389565 |
| STX8 | 0.881043917 | 0.881043917 |
| UBXN4 | 0.881768154 | 0.881768154 |
| SLC35E2B | 0.886775888 | 0.886775888 |
| CDC42BPG | 0.890286499 | 0.890286499 |
| BDKRB2 | 0.891675264 | 0.891675264 |
| BMPR2 | 0.893055182 | 0.893055182 |
| DVL2 | 0.893055182 | 0.893055182 |
| PPP4R1 | 0.893741825 | 0.893741825 |
| F2RL1 | 0.893741825 | 0.893741825 |
| GABRG2 | 0.897815239 | 0.897815239 |
| MAST3 | 0.899822092 | 0.899822092 |
| NPC2 | 0.908924249 | 0.908924249 |
| IGFBP3 | 0.910818321 | 0.910818321 |
| CD8A | 0.910818321 | 0.910818321 |
| TNFSF15 | 0.912692489 | 0.912692489 |
| MBL2 | 0.921764792 | 0.921764792 |
| SYTL5 | 0.923519545 | 0.923519545 |
| GIT1 | 0.925254395 | 0.925254395 |
| CADPS2 | 0.926969342 | 0.926969342 |
| FUBP1 | 0.928101583 | 0.928101583 |
| RHOT2 | 0.930339529 | 0.930339529 |
| CHKB | 0.93736849 | 0.93736849 |
| NCAPH | 0.938416698 | 0.938416698 |
| CPT2 | 0.938937485 | 0.938937485 |
| NCK2 | 0.93945606 | 0.93945606 |
| PROM1 | 0.941508247 | 0.941508247 |
| CECR5 | 0.944520185 | 0.944520185 |
| PRKCG | 0.947452513 | 0.947452513 |
| EVX1 | 0.948888823 | 0.948888823 |
| SSX2IP | 0.95030523 | 0.95030523 |
| TNFRSF11B | 0.951701734 | 0.951701734 |
| DDX42 | 0.954882845 | 0.954882845 |
| BVES | 0.955328444 | 0.955328444 |
| KLRG1 | 0.958385716 | 0.958385716 |
| NTF3 | 0.959662804 | 0.959662804 |
| EHD2 | 0.96050314 | 0.96050314 |
| PIGR | 0.963374654 | 0.963374654 |
| EIF2B2 | 0.963776024 | 0.963776024 |
| MORN4 | 0.964572131 | 0.964572131 |
| CPN1 | 0.965359393 | 0.965359393 |
| ADAT3 | 0.966907378 | 0.966907378 |
| ATF6 | 0.967668103 | 0.967668103 |
| AFF1 | 0.968045148 | 0.968045148 |
| ATXN10 | 0.969163015 | 0.969163015 |
| LRP11 | 0.969531215 | 0.969531215 |
| TESC | 0.970260979 | 0.970260979 |
| IGHG1 | 0.974788838 | 0.974788838 |
| MAL2 | 0.977389454 | 0.977389454 |
| NDUFB4 | 0.97770458 | 0.97770458 |
| HSPA13 | 0.979247038 | 0.979247038 |
| TNFSF14 | 0.982166097 | 0.982166097 |
| ALG10 | 0.985630268 | 0.985630268 |
| SELPLG | 0.987576307 | 0.987576307 |
| SLC15A2 | 0.988720711 | 0.988720711 |
| YIF1B | 0.992011287 | 0.992011287 |
| PRKG2 | 0.992198151 | 0.992198151 |
| GFRA2 | 0.992382804 | 0.992382804 |
| CD86 | 0.994107702 | 0.994107702 |
| ADAMTS4 | 0.994426145 | 0.994426145 |
| TMEM184C | 0.995471036 | 0.995471036 |
| CKAP2 | 0.995471036 | 0.995471036 |
| RNASEL | 0.996019465 | 0.996019465 |
| PLD3 | 0.996407567 | 0.996407567 |

**Supplementary Table 4 legend**

The table reports the results of the Seed Scores output from DAPPLE analysis. The score is calculated for each gene by enumerating the number of its connections and comparing this number to the values obtained in permuted networks (50,000 permutations) and the p-value is reported. A correction with Bonferroni is applied in case the sum of the connection is drawn from both the direct and the indirect interaction networks.

**Supplementary Figure 1 legend:**

The figure shows the workflow diagram to generate the analysis-ready dataset for single variant analysis on the Faroese samples. Given the limited size of the sample we decided to look only into those variants present in at least 3 individuals and, considering the use of an isolated population, we investigated only novel variants or those rare in outbred populations (MAF <0.05 in 1000 Genome CEU and GBR).
