# Supplementary figures and images for: Whole-exome sequencing of individuals from an isolated population implicates rare risk variants in bipolar disorder
Source: Transl Psychiatry. 2017 Feb 14;7(2):e1034–. doi: 10.1038/tp.2017.3 (PMC5438033; doi:10.1038/tp.2017.3)

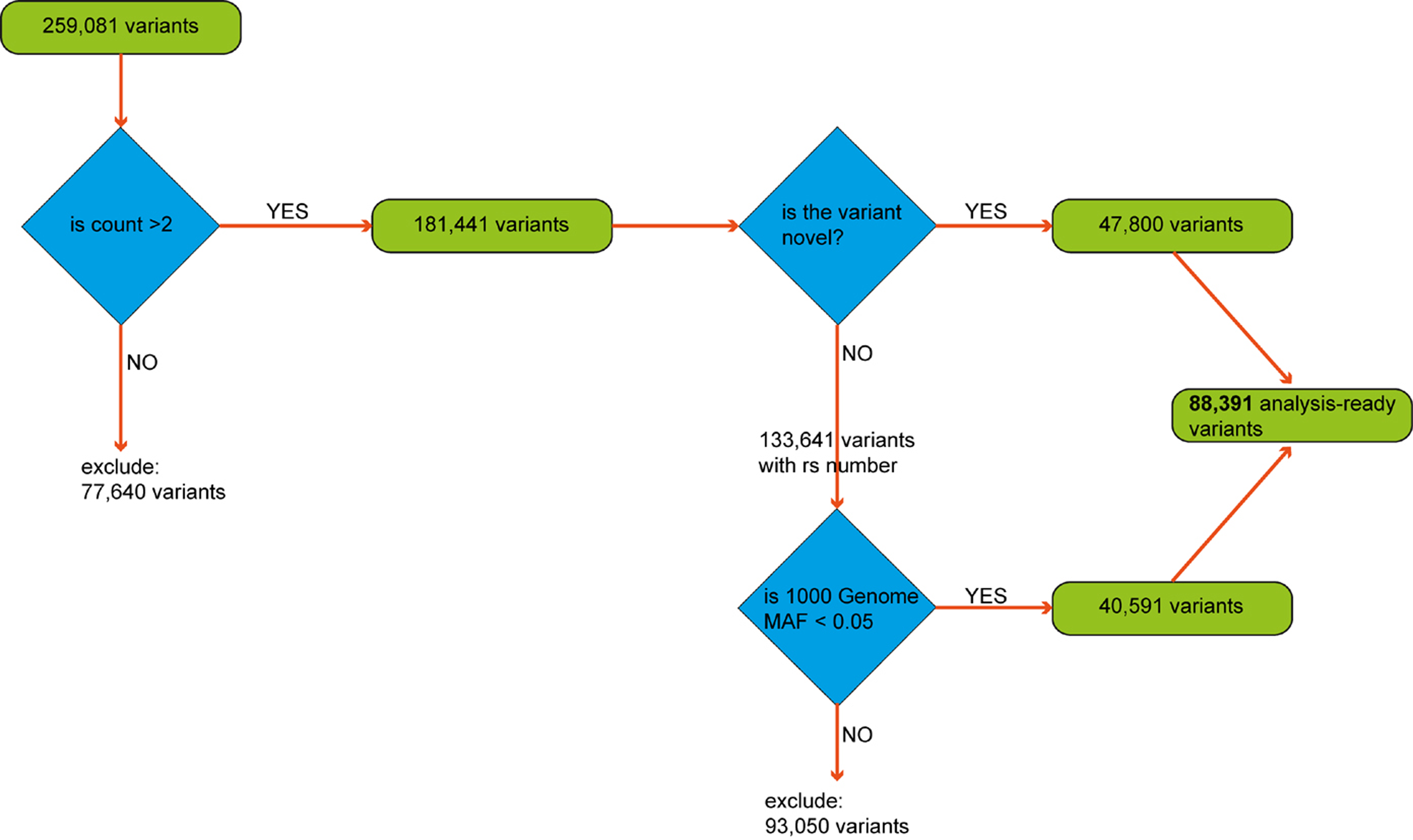

Supplement: Supplementary Figure [file tp20173x2.tif]
